# Vouchers: A Hot Ticket for Reaching the Poor and Other Special Groups With Voluntary Family Planning Services

**DOI:** 10.9745/GHSP-D-16-00084

**Published:** 2016-09-28

**Authors:** Elaine P Menotti, Marguerite Farrell

**Affiliations:** a United States Agency for International Development, Washington, DC, USA

## Abstract

Vouchers can be a highly effective tool to increase access to and use of family planning and reproductive health services, especially for special populations including the poor, youth, and postpartum women. Voucher programs need to include social and behavior change communication with clients and quality assurance for providers, whether in the private or public sector. In the longer term, voucher programs can strengthen health systems capacity and provide a pathway to strategic purchasing such as insurance or contracting.

## WHAT ARE VOUCHERS?

Vouchers are a form of results-based financing that have been used in many sectors, including the health sector, in low-, middle-, and high-income countries.^1^^,^^2^ Vouchers work as both financing mechanisms to ensure equity and programmatic tools to reduce barriers to access and increase use of critical health services. They are paper or electronic tickets that are distributed or sold to segments of the population who exchange them for health services at accredited facilities. To be accredited, a provider or outlet is generally reviewed against certain facility requirements and quality standards. When accompanied by social and behavior change activities and quality assurance approaches, including training, monitoring, supportive supervision, and site improvements, voucher programs can increase uptake of health services and improve service quality. A voucher program can also prepare a health system for strategic purchasing (e.g., insurance and contracting), engage the private sector, and protect the poor and other special groups. In the last decade, many countries in Asia and Africa have introduced medium- and large-scale voucher programs. We have an opportunity to learn from their experiences.

## HOW DO VOUCHERS WORK?

**FIGURE 1.**
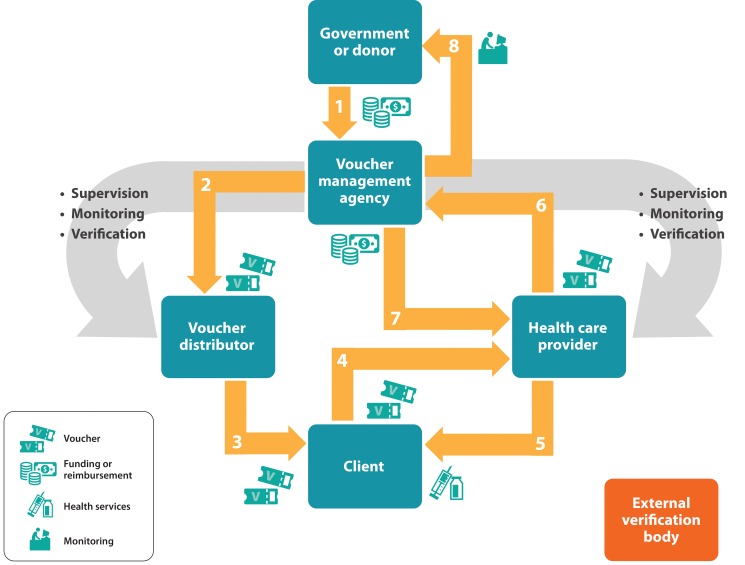
How Does It Work? A Flow Diagram of Voucher Transactions Source: Adapted from the World Bank 2005,^3^ Islam 2006,^4^ and Grainger et al. 2014.^1^

Once established, a voucher program involves a series of transactions between key players.^1^^,^^3^^–^^6^ Key transactions include the following, as illustrated by the numbered arrows in Figure 1:

1. A government or donor provides funding to the voucher management agency to establish and run systems and processes; identify and accredit providers and outlets to participate in the program; and provide training on voucher components and requirements to key players including providers and voucher distributors. In addition, the voucher management agency is engaged in monitoring and oversight of voucher distribution and service delivery on an ongoing basis.
2. The voucher management agency provides vouchers to trained distributors, such as NGOs and community health workers.
3. Voucher distributors counsel prospective clients and, if appropriate, give or sell vouchers (for a nominal amount) to a defined segment of the population, for example, the poor, youth, and pregnant or postpartum women.
4. Clients visit preapproved, quality-assured health care providers for products or services covered by the voucher program.
5. Providers give clients products or services free of charge in exchange for a voucher.
6. Providers then submit their claims to the voucher management agency for processing and reimbursement of services, according to a defined verification process.
7. The voucher management agency reimburses providers after verifying service provision.
8. The voucher management agency monitors, reviews, and examines data and submits reports to the donor/governance structure.

Some voucher programs also use an external verification body as an additional layer of monitoring outside of the role of the voucher management agency. This external verification body conducts audits at regular intervals to ensure vouchers are being used appropriately and provider claims are accurate.

**FIGURE 2.**
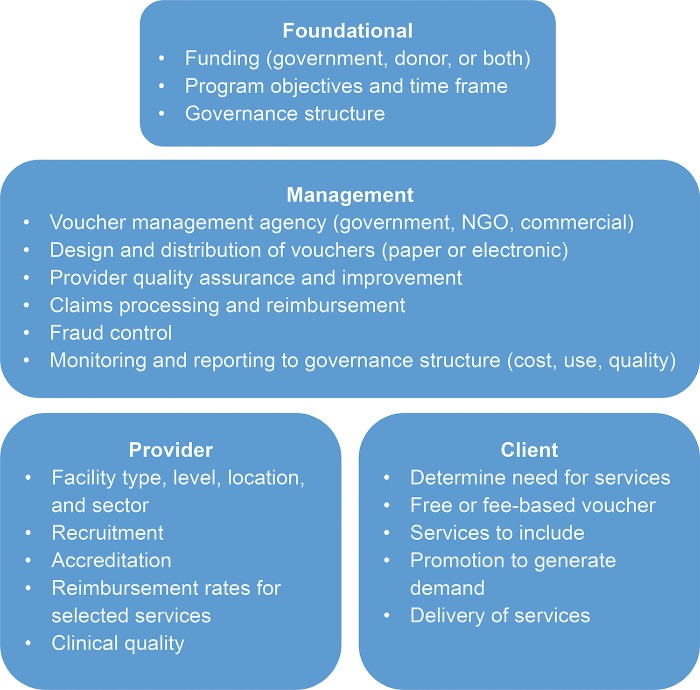
Key Features of a Voucher System

The objective of a voucher program is to address key barriers to accessing and using health services, especially among vulnerable populations. It should have the resources to operate at a medium to large level of scale, given the substantial systems investments required to set it up and operate. As outlined in Figure 2, the key design features of a voucher system cover 4 main areas:

- **Foundational** elements include the source of funding, generally donor or loan funds but could also be the government; program objectives and time frame; and the governance structure, often an advisory board comprised of government, donors, and other stakeholders.
- **Management** begins with identifying a capable voucher management agency (government, NGO, or commercial entity) that establishes systems and processes for selection and accreditation of health care facilities; provider quality assurance; designing, printing (if using paper vouchers), and delivering vouchers to distributors; claims processing and reimbursement; mitigation and control of fraud; and ongoing monitoring and reporting to the donor/governance structure. Alternatively, these functions could be executed by multiple agencies to leverage existing local capacity. Management of the voucher system may include external verification to ensure transparency and fraud mitigation. This is particularly common when the voucher management agency participates in service provision and implementation, for example, when social franchise organizations (typically NGOs) serve as the voucher management agency while using their franchised health clinic networks and existing contracts with health care providers to deliver services in voucher programs.
- **Provider** factors include determining the type, level, location, and sector (public, private for-profit, private not-for-profit, or a mix of these) of health care facility to include; recruiting, accrediting, and retaining qualified providers to participate in the voucher program; and identifying and establishing service packages and pricing. The design of the voucher program should incorporate ongoing quality assurance inputs and support to providers so that the services covered by the program achieve quality standards and revenue from vouchers can be reinvested into quality improvements.
- **Client** factors include identifying key segments of the population and their need for services, how much to charge for a voucher (always below the market rate) or whether to distribute the vouchers for free, how to distribute vouchers, which services to include, how to promote the services and the program to the intended population to generate demand, and how to deliver services. Vouchers may focus on a single health area, such as family planning, but could include multiple services in that area such as counseling, method provision, a follow-up visit, and method removal (as relevant). Alternatively, vouchers can include a package of services such as antenatal care, institutional delivery, postpartum services, child health, and family planning.

A voucher program should ideally have the resources to operate at a medium to large level of scale, given the systems investments required to set it up and operate.

Design of the voucher program should incorporate ongoing quality assurance inputs and support to providers so that covered services achieve quality standards and revenue from vouchers can be reinvested into quality improvements.

## THE ADVANTAGES OF A WELL-IMPLEMENTED VOUCHER PROGRAM

Voucher programs for family planning services have been implemented successfully in a range of settings where financial, information, and other barriers impede access to and use of modern contraceptives.^7^^,^^8^ For example, Box 1 illustrates how a voucher program in Uganda increased uptake of family planning services, particularly of voluntary long-acting reversible contraceptives, in private franchised clinics by removing barriers to access. Box 2 highlights two examples of voucher programs that provided family planning and reproductive health services for youth. A review of available literature and implementation experience shows that vouchers can be an effective programmatic tool as well as a financing mechanism for family planning and reproductive health.^2^^,^^7^^,^^9^^,^^13^^,^^14^ Key benefits and comparative advantage of voucher programs are summarized in the Table and outlined in more detail below.

BOX 1. **Voucher Program in Uganda Increases Access to and Use of Long-Acting Reversible Contraceptives**

From 2011 to 2014, Marie Stopes Uganda implemented a voucher program in private franchised clinics to provide family planning services to more than 325,000 clients; 66% were not using a contraceptive method before the program and nearly 80% had no education or only primary education (a proxy indicator for low income).^9^

The voucher program offered all methods of contraceptives; however, the majority (94.9%) chose long-acting reversible contraceptives, which have limited availability in the public sector and may be too expensive for some clients. Estimates show that the voucher program increased modern contraceptive prevalence by nearly 1.5 percentage points. It is clear that reducing financial and other access barriers successfully increased uptake of family planning services.

BOX 2. **Spotlight on Voucher Programs for Youth**

In Madagascar, a voucher program implemented between July 2013 and December 2014 provided family planning and reproductive health services for more than 43,000 youth who faced financial and other barriers in accessing a full range of services; 78% chose an implant or intrauterine device, and just over half received screening or treatment for sexually transmitted infections.^10^

In Nicaragua, a youth voucher program running from September 2000 to July 2001 that provided a total of 28,771 vouchers, found that youth participating in the program were 3 times more likely to use family planning and reproductive health services, 2 times more likely to use modern contraception, and 2.5 times more likely to report condom use at last sexual contact compared with youth not participating in the program.^11^ Researchers also found that providers participating in the Nicaragua voucher program had better knowledge, improved practices, and some attitudinal changes in support of provision of family planning and reproductive health services to youth compared with providers not involved in the program.^12^

**TABLE:** Key Advantages and Challenges of Voucher Programs

Advantages	Challenges
Reduce financial and other client barriers to accessing health services.	Require establishment of systems and processes to execute and monitor program.
Allow governments and donors to target subsidies for populations in need, such as poor, youth, and pregnant or postpartum women.	Can be complex to target to defined groups, particularly for groups new to vouchers. Requires measures to mitigate fraud and leakage to general population or non-poor.
Allow resources to be directed toward key or high-impact health interventions and can stimulate demand for health services and behaviors.	Unclear how they may affect other health services and the overall health system; could result in provider overload if the system is not prepared.
Create a network of quality-assured health care providers, which can enhance access to services in the short term, and a platform for strategic purchasing in medium to longer term.	Require quality inputs to service providers to improve quality of care; quality inputs also prevent driving up service use without improving health outcomes and client satisfaction.
Voucher revenue can flow directly to health care providers, which the providers can then reinvest in facilities and services to further improve them.	Program funding may or may not be used to invest in areas that improve services or client satisfaction.

- **Target health care subsidies toward the poor and other special groups.** Vouchers, a demand-side financing mechanism by design, enable governments and donors to target subsidies directly for populations in need, including poor, youth, and pregnant or postpartum women, rather than spreading limited resources among the general population. Vouchers make sense as a solution when there are financial and other access barriers to seeking and using key high-impact health services. Most programs use means testing or geographic targeting to identify poor or otherwise vulnerable clients to channel resources toward those most in need of financial subsidy.^1^ Recent improvements in standardized equity measurement can simplify this process and reduce the cost in voucher programs.^15^
- **Promote demand for and use of family planning and reproductive health services while enhancing client choice.** Voucher programs can be a promotional tool to expand access to and choice of health care services.^16^ Programs can and should accompany voucher distribution with social and behavior change communication efforts to promote demand for and improve knowledge of the key health behaviors and services.^5^ Promotion of the voucher program helps raise client awareness of what services are offered and where, particularly if they are new or underused services; without promotion efforts, clients may not be aware of and use the voucher.^6^^,^^16^ In one voucher program area, only 25% of women from the communities had heard of the family planning voucher, versus 82% who had heard of the safe motherhood voucher; corresponding family planning service uptake was lower than for safe motherhood. Researchers suggest that low uptake may have been due in part to the absence of adequate communication with clients and communities about the family planning voucher program.^17^^,^^18^ Vouchers can particularly address access barriers for clinical services, such as long-acting and reversible contraceptives and permanent methods, in areas where poor women are less likely to use them, by reducing out-of-pocket costs for clients and ensuring providers get reimbursed for these methods that are relatively costlier to provide than short-acting methods.^5^^,^^13^^,^^19^^–^^21^ Often, providers are motivated to participate in a voucher program as it gives them an opportunity to gain new skills, offer new services to clients, and make their clinic more of a “one-stop shop” for clients.^22^ Because vouchers subsidize the poor’s purchasing power, this can facilitate the poor’s access to the private sector without spending money out of pocket, thereby reducing inequities in access to health services including family planning.^12^^,^^23^
- **Engage and leverage the existing health system to maximize service delivery.** Voucher programs can be designed to fit the existing health system and mix of providers, although most programs engage both private- and some public-sector providers to ensure maximum reach and to leverage capacity.^1^ Engaging the private sector in voucher provision is particularly important in contexts where quality may be weak in the public sector, where people seek health care in the private sector, and use of preventive services is limited.^12^ Increasing the provider mix in a network also extends the reach of social protection and creates more provider choice for clients.^12^^,^^24^^,^^25^ In addition, voucher programs can establish a network of health care providers from fractionalized separate entities that were not linked before, but are now accredited voucher service providers.^13^ A voucher program in Yemen, implemented by an NGO, kept primary health services, including family planning, running in public clinics or offered the choice of private clinics when the government was unable to flow resources due to active conflict.^26^ A successful voucher program implemented by private franchised clinics in Pakistan expanded access to family planning services, as none of the providers offered these services before participating in the voucher program.^13^ Providers often like participating in a voucher program because they can increase the number of clients served and attract new clients while offering a range of services, potentially making clinics more profitable.^3^^,^^5^^,^^12^
- **Assure and improve the quality of family planning and reproductive health care.** Implicit in a voucher program is that participating health care facilities must be accredited and/or provide services according to established quality standards. Programmatic inputs are often required to get a facility accredited and enrolled in the voucher program and fully functional within the system to process voucher claims. Ongoing quality assurance and provider support can also be a feature of the design, and is a key component when implemented by private social franchise clinic providers. Reimbursements given to providers in exchange for provision of services can then be channeled toward facility-level improvements (e.g., supplies and commodities, staff, equipment, infection prevention, and training). In Kenya, both public and private providers invested voucher revenue into facility-based quality improvements.^17^ Finally, providers know that clients can choose any provider participating in the voucher program, thereby driving competition and encouraging providers to make quality improvements, such as privacy, cleanliness, and the addition of other services.^27^ In Kenya, postnatal clients at facilities participating in the voucher program received more comprehensive counseling on fertility, healthy birth spacing, and available contraceptive methods than postnatal clients at comparable non-participating facilities.^28^ In the longer term, vouchers can institutionalize performance in a clinical setting by focusing attention on quality services rendered, and for public-sector clinics this can improve productivity and efficiency.^29^
- **Provide a pathway for health care providers to participate in insurance.** Most voucher programs are financed by donors or loan funds, but these could eventually be financed by governments with domestic resources. Vouchers can be a precursor to health insurance or government contracting, familiarizing providers with processes like accreditation, standardization of service packages and pricing, billing, verification, and reimbursement after service provision.^24^^,^^30^ Vouchers demand provider-level accountability and, when implemented well, they create disincentives for fraudulent behavior. In Armenia, a voucher program was developed to prevent informal payments and strengthen accountability.^1^ By linking qualified providers under a program umbrella, voucher programs can organize a fractionalized private sector or a mix of public and private health care facilities into a quality-assured network, which could then provide services for government health programs or insurance. In Cambodia, Kenya, Tanzania, and Uganda, voucher programs were introduced to build social health insurance capability in the health sector and to increase government’s familiarity with purchasing services from the private sector.^1^ Successfully contracting with the private sector in a voucher program resulted in a government plan for how to scale up the program nationally.^1^ Voucher programs that work with social franchise clinics can network fractionalized private providers with a third-party intermediary organization (usually an NGO) that addresses quality inputs, simplifies reimbursement, and lends itself to future financial transaction flows from governments to multiple private providers.

Voucher programs help to protect vulnerable groups, increase uptake of health services, improve service quality, engage the private sector, and strengthen health systems.

The private sector serves an especially important role where public-sector quality may be weak, where people already seek private care, and where use of preventive services is limited.

Voucher programs can organize health care facilities, including a fractionalized private sector, into a quality-assured network, which could then provide services for government health programs or insurance.

## THE CHALLENGES OF IMPLEMENTING VOUCHER PROGRAMS

The potential for voucher programs are substantial, but challenges in their implementation do exist. As with most complex interventions, implementers must focus on the specifics and get immersed in the details to ensure a well implemented program. With experience, the design and function of the voucher system improves and implementation becomes easier. Vouchers can work in a range of contexts, but program implementation should be iterative and dynamic, with monitoring and regular opportunities to adjust designs, service packages, pricing, methods of means testing to identify populations in need, and voucher distribution approaches to ensure optimal functioning and to reach the defined client population.^1^ The following questions are important to consider:

- **Programs require substantial administration and oversight—do the benefits outweigh the challenges?** Voucher programs are somewhat complex to set up and manage, and they require ongoing oversight and management inputs. Because of this, voucher programs should be implemented at a medium to large scale to maximize investment in these systems. Costs are high at the early stages of setting up systems, but they should decrease over time as systems are established and expertise is strengthened.^6^ Using local organizations to manage voucher programs can lower administrative costs.^24^ In some countries, vouchers relied on existing ways to identify the poor and to target benefits, such as in India’s Below Poverty Line card or Cambodia’s Health Equity Fund.^1^^,^^5^
- **How can public-private partnerships be encouraged?** The majority of voucher programs to date have been designed to facilitate contracting of health services through the private sector, including social franchise clinics,^1^ to leverage private-sector reach, expand provider choice, and improve quality service provision. Voucher programs can, however, function as a type of public–private partnership, with specific roles and functions carried out by the government and private sector. For example, in India a formal public–private partnership approach to the Sambhav voucher program was considered a success.^5^ In many countries, public-sector health care services are mandated as free, so governments may face difficulty in making the rationale to include public-sector services in a voucher program. Without concerted efforts to address operational challenges, governments may not have the infrastructure, staff, or systems in place to adequately implement, oversee, and manage all aspects of a voucher program to function optimally.^25^ However, even if public-sector health care provision and facilities are not included, governments can play important roles in oversight, planning, and priority setting with the longer-term view of transitioning a voucher program to government financing, contracting, or insurance. While vouchers should not compete with other social protection mechanisms, such as social health insurance, they should be positioned as complementary and a partnership opportunity to stimulate uptake of health services and incentivize high-quality service provision. Vouchers can also fill a gap when family planning methods or services are not covered, or only partially covered, by insurance packages.
- **How does voucher programming influence service provision as a whole?** While vouchers may play an important role in ensuring access to and delivery of key health services to populations in need, it is not clear how vouchers affect service provision as a whole or how they affect services not covered by vouchers or clients without vouchers.^25^ Voucher programs provide facility-level support (e.g., improving infection prevention measures; ensuring adequate clean water/sanitation facilities; installing adequate client privacy measures; training on clinical standards and guidelines; ensuring availability of high-quality commodities, instruments, and consumables; providing job aids, counseling guides, and client informational materials) to ensure that providers have sufficient quality standards to participate in the program, which may have spillover effects on improving the quality of other health services (e.g., infection prevention or client-centered care). In addition, revenue from voucher programming may enable health care facilities to hire staff or purchase supplies and equipment to add or improve services, potentially improving specific services, the client experience, or health care provision overall. However, it is also possible to increase uptake of services but not see any improvements in outcomes or service quality, as shown by researchers examining maternal health demand-side financing programming, including vouchers.^31^ For family planning, this may not be the case, as any use of services can improve overall health outcomes, such as maternal and infant mortality reduction, but it is important to provide high-quality services and continuity of care with vouchers. In addition to quality assurance inputs and training of providers, a family planning voucher program in Uganda aimed for continuity of care by offering a single voucher that included 4 services with separate, reimbursable barcodes: family planning counseling, method provision, a follow-up visit to address any side effects, and a removal visit (for long-acting and reversible contraceptives).^8^
- **Should family planning be integrated into a multi-service, integrated voucher program or have its own voucher?** In Uganda, single-service family planning vouchers have operated with success, including alongside other types of health vouchers.^9^^,^^13^^,^^14^^,^^21^ Most voucher programs, however, offer integrated services, such as a program in India that included a package of antenatal, institutional delivery, postpartum, and family planning services.^1^ In Zimbabwe, a voucher program for youth initially included only family planning services, but later added screening and treatment for sexually transmitted infections after receiving feedback from clients who were interested in and needed more services (personal communication with Anna Mackay, Deputy Director, Support for International Family Planning Organizations [SIFPO-MSI] Project, Feb. 2016). It is unclear exactly the best way to include family planning services in a bundled package so that the family planning services are still promoted and provided. Experience from two different voucher programs in different areas of Pakistan suggests that a separate family planning visit may yield more uptake.^32^^,^^33^ In one of these programs, 79% of voucher clients visited clinics for family planning services when the services were offered through a separate postnatal care visit. In comparison, 62% of clients in the second program visited clinics for family planning as part of a package of postnatal care services.
- **How can we prepare providers to handle an increase in demand for services?** A key design feature of voucher programs to ensure success is the appropriate selection and retention of qualified health care providers. Retaining qualified providers is particularly important for programs that provide resource-intensive quality monitoring and supervision. Voucher programs may stimulate rapid uptake of services, and if providers are not prepared (e.g., with adequate staff, commodities, and supplies; sufficient operating hours; efficient management of services) the increased service volume may have negative consequences on provider job satisfaction.^22^ The increased demand for services may also result in providers not being able to provide comprehensive or client-centered care due to time constraints. The possibility of overload may be more acute in public-sector health care facilities, which may have less ability to hire staff, extend hours, or use the reimbursement revenue to make changes, unless the program is designed to address these challenges.^25^ Private-sector clinics may have more autonomy over the revenue to direct it toward quality improvements, whereas public-sector clinics may not have the autonomy to organize service provision or direct access to resources flowing into the facility.^1^

Costs may be higher at the early stages of setting up voucher systems but should decrease over time as systems are established and expertise is strengthened.

Single-service family planning vouchers have been successful, but most voucher programs offer a package of integrated services.

Regular monitoring, including course corrections, is a key component of voucher program success.

## NEXT STEPS

When implemented at a medium to large scale, voucher programs can achieve results, including reducing barriers to access and improving uptake of key health services to meet the health needs of the poor, vulnerable, and other special populations, in part because of their aim and perhaps ability to facilitate change at both client and provider levels. Voucher programs may also get populations familiar with using key preventive and essential primary health care services and thus may change behavior at a social normative level, another way of ensuring sustainability.

As we work to reaching the Family Planning 2020 (FP2020) goals by addressing gaps in access to family planning, vouchers offer an opportunity to target donor and government resources to those who most need family planning services and products. Those with the highest unmet need may not seek family planning services without subsidies or support and are therefore vulnerable to unintended pregnancy and adverse maternal health outcomes. As we look to improve access to family planning for 120 million more women and girls, we should focus donor and government resources on ensuring that the poor and other special groups can achieve their reproductive intentions.

Vouchers can support the aims of FP2020--addressing gaps in family planning access by targeting resources to those who want voluntary family planning services but who may not otherwise seek services without a subsidy or support.*

While universal health coverage may be a vision for the future, there is less discussion in the literature on which short- or medium-term steps we need to take to get there. Vouchers could serve as an intermediate step for public financing to begin to target subsidies to address inequitable rates of maternal, child, and infant mortality among the poor, versus traditional supply-oriented health financing that faces challenges in reaching the poor and underserved.^3^^,^^4^^,^^16^ Voucher programs may strengthen capacity and readiness in the health system for implementing universal health coverage that prioritizes the health needs of the poor, engages the private sector, and has a service package that does not leave out key primary health interventions and preventive care, such as family planning. In Cambodia, a voucher program became better aligned with the government’s Health Equity Fund, a safety net program for the poor,^1^ and even helped to identify new beneficiaries. However, such experiences of transitioning voucher programs into larger health insurance programs are limited.

We need partner organizations to document their experiences with vouchers, share their results, and demonstrate impact through research when possible.

The community of practice on health voucher implementation in developing countries is growing and has experiences to share, as many voucher programs must undergo midcourse corrections and adaptions to function optimally. We need partner organizations to document their experiences and share their results, and conduct research when possible to further demonstrate impact and contribute to the body of knowledge to address common challenges and issues in implementation. We have more to learn about voucher programs, and we encourage the implementation and research communities to collaborate to shed light on the following areas:

1. Understand key differences in voucher programs that are implemented by public-sector, private-sector, or mixed public- and private-sector health care facilities, and ways to maximize performance, particularly how best to strengthen implementation and quality in public-sector voucher programs.
2. Gain efficiencies in voucher program operations including:
  Best practices for provider reimbursement
  Opportunities for leveraging mobile health technology (e.g., electronic vouchers, provider reimbursement using mobile money, applications for tablets or mobile phones to facilitate provider supervision and oversight techniques)
  Optimal strategies to target voucher subsidies toward the populations that can most benefit from them while minimizing fraud
  Strengthened monitoring of programs with aligned key outcomes of interest
3. Document more experiences of medium- to large-scale voucher programs that provide information on performance (e.g., client uptake, new or lapsed family planning clients, poverty status of voucher clients, and observed and reported quality) and cost.
4. Test and document the transition of a donor-funded voucher program to government financing.

## Notes

**Peer Reviewed**

**First Published Online:** 2016 Sep 26
